# Element of Surprise? Rice as a Source of Arsenic in Children’s Diets

**DOI:** 10.1289/ehp.120-a402b

**Published:** 2012-10-01

**Authors:** Rebecca Kessler

**Affiliations:** Rebecca Kessler, based in Providence, RI, writes about science and the environment for various publications. She is a member of the National Association of Science Writers and the Society of Environmental Journalists.

Last winter, the discovery of arsenic in foods containing organic brown rice syrup, including toddler formulas, made headlines. Members of the same research team now report higher urinary arsenic concentrations in children who eat any type of rice than in children who don’t, suggesting this food may be an important source of arsenic exposure for children in the United States [*EHP* 120(10):1418–1424; Davis et al.].

Rice is an economic, nutritious staple food consumed around the world, and its popularity is growing in the United States. In addition, rice flours, syrups, and other products are widely used in processed foods. But rice plants are especially well suited to accumulating arsenic from the soil in which they grow. The arsenic content of rice varies widely depending on where it was grown and how it was processed.

In the current study, researchers examined data for 2,323 children who participated in the National Health and Nutrition Examination Survey between 2003 and 2008. They compared concentrations of arsenic in the urine of children estimated to have eaten at least a quarter cup of cooked rice during the previous 24 hours and the urine of children estimated to have eaten none. These estimates were based on children’s consumption of rice itself plus foods that contain rice-based ingredients.

“Rice eaters” had a median urinary arsenic concentration of 8.9 μg/L compared with 5.5 μg/L for “non-rice eaters.” After adjusting for possible confounding factors and excluding children who had eaten seafood (a major source of a form of arsenic that is considered nontoxic) during the past 24 hours, the study found that urinary arsenic concentration increased 14.2% with every quarter-cup increase in rice that the children ate. The association was greater in children aged 6–11 years than in children aged 12–17 years, suggesting possible metabolic or dietary differences between the two age groups.

One limitation of this study is that the researchers assessed all forms of rice combined and did not examine the impact of specific types of rice on urinary arsenic concentration. Another is that estimates of the rice content in each processed food may have been off.

There is some evidence that high levels of arsenic exposure during childhood are associated with neurobehavioral problems as well as cancer and lung disease later in life. However, further research is needed to understand the health effects of exposures like those observed in this study.

